# Time course of changes in the range of motion and muscle-tendon unit stiffness of the hamstrings after two different intensities of static stretching

**DOI:** 10.1371/journal.pone.0257367

**Published:** 2021-09-14

**Authors:** Kosuke Takeuchi, Kazunori Akizuki, Masatoshi Nakamura

**Affiliations:** 1 Department of Physical Therapy, Kobe International University, Kobe, Hyogo, Japan; 2 Institute for Human Movement and Medical Sciences, Niigata University of Health and Welfare, Niigata, Japan; Universita degli Studi di Milano, ITALY

## Abstract

**Objectives:**

The purpose of this study was to examine the time course of changes in the range of motion and muscle-tendon unit stiffness of the hamstrings after two different intensities of static stretching.

**Methods:**

Fourteen healthy men (20.9 ± 0.7 years, 169.1 ± 7.5cm, 61.6 ± 6.5kg) received static stretching for 60 seconds at two different intensities based on the point of discomfort (100%POD and 120%POD) of each participant, in random order. To evaluate the time course of changes in the flexibility of the hamstrings, the knee extension range of motion (ROM), passive torque at end ROM, and muscle-tendon unit stiffness were measured pre-stretching, post-stretching, and at both 10 and 20 minutes after static stretching.

**Results:**

For both intensities, ROM and passive torque at pre-stretching were significantly smaller than those at post-stretching (p < 0.01 in both intensities), 10 minutes (p < 0.01 in both intensities), and 20 minutes (p < 0.01 in both intensities). The muscle-tendon unit stiffness at pre-stretching was significantly higher than that at post-stretching (p < 0.01), 10 minutes (p < 0.01), and 20 minutes (p < 0.01) only in the 120%POD, but it showed no change in the 100%POD.

**Conclusion:**

The results showed that ROM and passive torque increased in both intensities, and the effects continued for at least 20 minutes after stretching regardless of stretching intensity. However, the muscle-tendon unit stiffness of the hamstrings decreased only after static stretching at the intensity of 120%POD, and the effects continued for at least 20 minutes after stretching.

## Introduction

Static stretching is used to increase flexibility and prevent injuries [1,2], and range of motion (ROM) is often measured as an indicator of flexibility. Previous studies pointed out that ROM was affected by both stretching tolerance [3–6] and muscle-tendon unit stiffness [7–10]. The muscle-tendon unit stiffness is calculated from the torque-angle curve during passive joint movement and it reflects the viscoelasticity of the muscle-tendon unit [7–10]. Previous studies reported that muscle-tendon unit stiffness is involved in the occurrence of sports-related injuries, such as muscle strain [11] and achilleas tendon injury [12]. Static stretching effectively decreases the muscle-tendon unit stiffness [3,13]. Therefore, a previous systematic review study recommended to use static stretching as part of a fundamental warm-up routine to prevent sports-related injuries [14].

The effect of static stretching on the muscle-tendon unit stiffness is affected by the intensity [15–19] or duration of the stretching [7,8,20]. The intensity of static stretching is determined based on the ROM [15,16,19] or point of discomfort (POD) [17,18,21] of each participant, and static stretching at the intensity of 100%POD is the normal intensity, which is performed at the maximum joint angle without pain. When static stretching is performed at the intensity of 100%POD, 180 seconds of static stretching is needed to decreased the muscle-tendon unit stiffness of the hamstrings [7,8], which is the most common site of muscle strain [22–25]. On the other hand, high-intensity static stretching has been reported as a new technique to effectively decrease the muscle-tendon unit stiffness of the hamstrings in a shorter stretching duration [15,17,18]. Previous studies reported that [26,27] high-intensity static stretching at the intensity of 120%POD or more decreased the muscle-tendon unit stiffness of the hamstrings to a greater extent compared to static stretching at the intensity of 100%POD, even if the duration of the stretching was for less than 60 seconds.

Several previous studies investigated the time course of changes in the flexibility of the hamstrings [28] and triceps surae after static stretching at the intensity of 100%POD [9,28–32]. It is useful for clinicians and athletes to know the time course of changes in flexibility after static stretching [33]. Clinicians often use static stretching for their athletes with the expectation that an improvement of flexibility after stretching will last long enough to have at least a temporary beneficial effect. It was reported that there was an increment in ROM and decrement in the muscle-tendon unit stiffness after static stretching at an intensity of 100%POD, and the changes returned to the baseline level within 30–60 [10,28] and 5–20 minutes [28–32], respectively. Therefore, it was suggested that the change in the muscle-tendon unit stiffness after static stretching at the intensity of 100%POD disappeared more rapidly than the change in ROM [10,28,29]. It is possible that high-intensity static stretching (120%POD) has longer lasting effects on any decrease in the muscle-tendon unit stiffness compared to normal-intensity static stretching (100%POD) because high-intensity static stretching has a large effect on the decrease in the stiffness. However, the time course of changes in the flexibility after high-intensity static stretching has not been investigated. It is necessary to examine the time course of changes in the flexibility of the hamstrings after high-intensity static stretching to utilize it effectively.

The purpose of the present study was to examine the time course of changes in the range of motion and muscle-tendon unit stiffness of the hamstrings after 1 minute of different intensities of static stretching (100%POD and 120%POD). It was hypothesized that the decrease in the muscle-tendon unit stiffness of the hamstrings would disappear within 20 minutes after 1 minute of high-intensity static stretching, based on previous studies [28–32]. To investigate the time course of change in the muscle-tendon unit stiffness in detail, the flexibility of the hamstrings was measured pre-stretching, post-stretching, and at both 10 and 20 minutes after static stretching.

## Methods

### Procedure

A randomized crossover trial was conducted. Participants underwent two different intensities (100%POD and 120%POD intensities) of static stretching in their right hamstrings, in random order. 120%POD intensity was chosen because previous studies [17,18] showed that static stretching at the intensity of 120%POD significantly decreased the muscle-tendon unit stiffness of the hamstrings. The participants visited two times on separate days, with an interval of one week between visits. Participants attended a familiarization session 1 week before the first testing day. To evaluate the time course of changes in the flexibility of the hamstrings, the knee extension ROM, passive torque at end ROM, and muscle-tendon unit stiffness were measured pre-stretching, post-stretching, and at both 10 and 20 minutes after static stretching. In addition, to investigate the pain in the hamstrings, a numerical rating scale (NRS) was examined during static stretching and after the post-stretching measurement. The experiment was performed in an university laboratory, where the temperature was maintained at 25°C.

### Participants

Fourteen active men (20.9 ± 0.7 years, 169.1 ± 7.5cm, 61.6 ± 6.5kg) were recruited. Participants who were competitive athletes, who performed regular intensive stretching practice or strength training, or those who had a history of lower limb pathology were excluded. The sample size of the muscle-tendon unit stiffness was calculated with a power of 80%, alpha error of 0.05, and effect size of 0.25 (middle) using G*Power 3.1 software (Heinrich Heine University, Düsseldorf, Germany), and the results showed that the requisite number of participants for this study was 12 participants; thus, 14 participants were recruited to account for possible attrition. All participants were informed of the requirements and risks associated with their involvement in this study and signed a written informed consent document. The study was performed in accordance with the Declaration of Helsinki (1964). The Ethics Committee of Kobe International University approved the study (Procedure #G2020-160).

### Flexibility assessment

**Fig 1:**
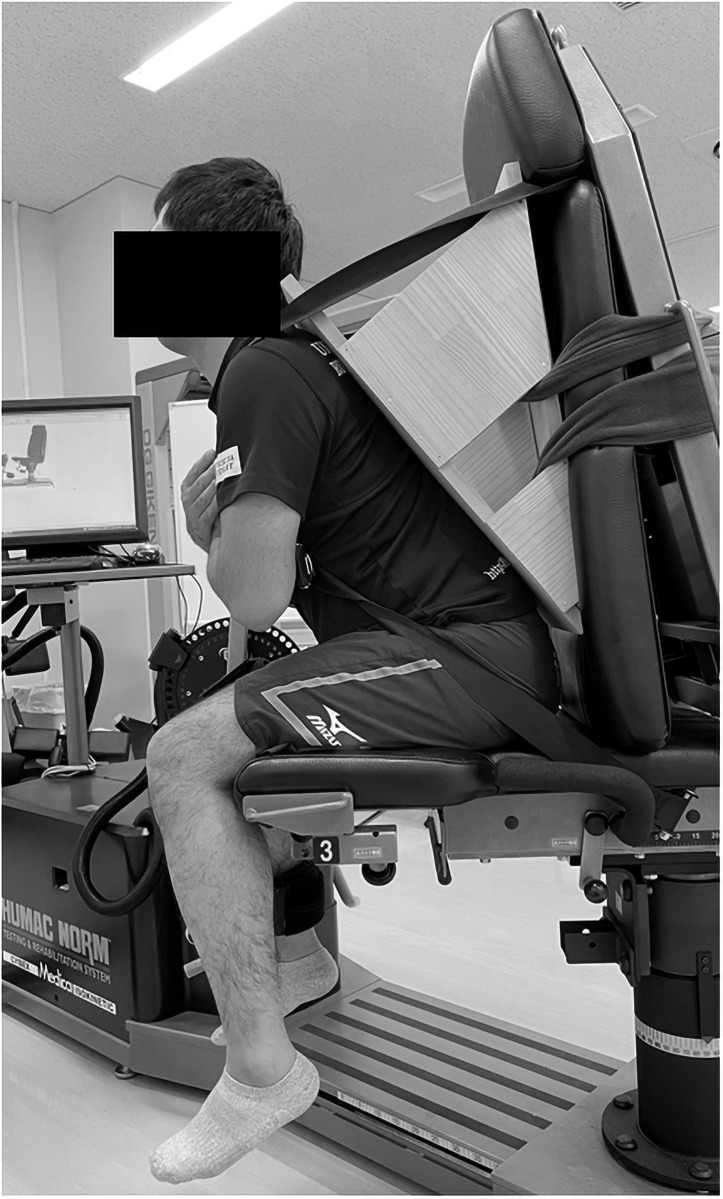
Experimental setting.

The flexibility assessment was performed in the same fashion as previous studies [15,17,18]. In the present study, an isokinetic dynamometer machine (CYBEX NORM, Humac, California, USA) was used. This study used a sitting position in which the hip joint was flexed, which has been shown to efficiently stretch the hamstrings (Fig 1) [15]. The participants were seated on a chair with the seat tilted maximally, and a wedge-shaped cushion was inserted between the trunk and the backrest, which set the angle between the seat and the back at approximately 60 degrees. The chest, pelvis, and right thigh were stabilized with straps. The right knee joint was aligned with the axis of the rotation of the isokinetic dynamometer. The lever arm attachment was placed just proximal to the malleolus medialis and stabilized with straps. In the present study, reported knee angles were measured using the isokinetic dynamometer. A 90-degree angle between the lever arm and floor was defined as 0 degrees of knee flexion/extension. The flexibility assessment was performed before static stretching (pre-stretching), immediately after stretching (post-stretching), and at both 10 and 20 minutes after stretching. The participants rested on the dynamometer at 0 degrees of the knee joint between measurements. The participants were instructed to relax during the flexibility assessment.

The effects of the flexibility measurement maneuver on the subsequent flexibility measurement were examined with 8 participants. They rested on the isokinetic dynamometer machine for 90 seconds. The flexibility measurements were performed pre-rest, post-rest, and at both 10 and 20 minutes after the rest period. A one-way repeated ANOVA showed that there was no significant difference between the time (pre-rest, post-rest, 10 minutes, and 20 minutes) in the knee extension ROM (p = 0.99), passive torque at end ROM (p = 0.83), or muscle-tendon unit stiffness (p = 0.78).

### Knee extension ROM, passive torque at end ROM, and muscle-tendon unit stiffness

The knee extension ROM and passive torque were measured by using the isokinetic dynamometer. The knee joint was passively extended from 0 degrees to the maximum angle without pain at 5 degrees/second. A previous study showed that the velocity does not cause a stretch reflex [34]. The knee extension ROM was defined as the range from 0 degrees to the maximum knee extension angle. The passive torque during the knee extension ROM measurement was recorded in the isokinetic dynamometer. After the experiment, the knee extension angle and passive torque during the flexibility measurement were exported to a personal computer, and the passive torque and muscle-tendon unit stiffness were analyzed. The passive torque at the maximal knee extension angle (end ROM) was used for further analyses.

The muscle-tendon unit stiffness of the hamstrings was defined as the values of the slope of the regression line that was calculated from the torque-angle curve using the least-squares method [15,18,35]. The muscle-tendon unit stiffness was calculated from the same knee extension angle range before and after static stretching. The calculated knee extension angle range was defined as the angle from the 50% maximum knee extension angle to the maximum knee extension angle measured before static stretching [7,15,18]. However, if the maximum knee extension angle measured after static stretching was smaller than that before stretching, the muscle-tendon unit stiffness was calculated from the 50% maximum knee extension angle to the maximum knee extension angle measured after stretching [15,18].

### Numerical rating scale

The level of pain during static stretching (repetitions 1 and 2) and after static stretching was quantified by an 11-point NRS that ranged from 0 (no pain) to 10 (worst imaginable pain) [15,18]. NRS was assessed 15 seconds after the start of each static stretching intervention and 1 minute after the end of the stretching intervention.

### Static stretching

All variables except NRS were described as mean ± SD in the present study, NRS was described as a median (interquartile range). Static stretching was performed in the same fashion as previous studies [17,18]. The participants were secured on the isokinetic dynamometer in the same fashion as the measurement of the knee extension ROM. The knee joint was passively extended from 0 degrees to the target intensities (100%POD and 120%POD). This position was then held for 30 seconds. This procedure was repeated two times, with intervals of 30 seconds, that is, a total of 60 seconds of static stretching was performed. The present study used a constant angle stretching procedure. Static stretching was performed at two different intensities based on the POD of each participant (100%POD and 120%POD). At 100%POD intensity, the angle was set just prior to the POD. At 120%POD intensity, the angle was set to 1.2 times the POD. The participants were instructed to relax during each static stretch.

### Reliability

The test-retest reliability for all dependent variables was determined in 8 males (21.1 ± 0.7 years, 170.9 ± 6.7cm, 61.9 ± 4.5kg). The 2 tests were separated by 3 days and were performed at the same time of the day. The reliability of knee extension ROM (intraclass correlation coefficient (ICC) of 0.97), passive torque at end ROM (ICC of 0.96), and muscle-tendon unit stiffness (ICC of 0.89) were acceptable in this study.

### Statistical analyses

For the knee extension ROM, passive torque at end ROM, and muscle-tendon unit stiffness, a two-way repeated measures ANOVA was used to examine the effects of intervention (100%POD vs. 120%POD) and time (pre-stretching vs. post-stretching vs. 10 minutes vs. 20 minutes). For NRS, a two-way repeated-measures ANOVA was used to examine the effects of intervention (100%POD vs. 120%POD) and time (first repetition vs. second repetition vs. post-stretch). If a significance was detected, post hoc analyses using Bonferroni’s test were performed. The analyses were performed using SPSS version 25 (SPSS, Inc., Chicago, IL, USA). Differences were considered statistically significant at an alpha of 0.05. To describe the effect size, the partial eta squared value was calculated by using the SPSS software.

## Results

### Knee extension ROM

**Fig 2:**
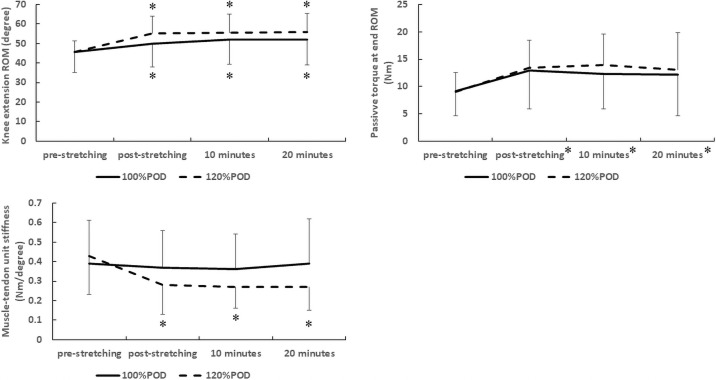
Time course of changes in flexibility after static stretching * p < 0.01 vs. value at pre-stretching in the same intensity. ROM: Range of motion.

There was a significant main effect for time (p < 0.01, partial eta squared = 0.66, F = 50.47) but no main effect for intervention (p = 0.44, partial eta squared = 0.02, F = 0.62). There was a significant two-way interaction effect (intervention × time, p < 0.01, partial eta squared = 0.14, F = 4.17) (Fig 2). For both intensities, the knee extension ROM at pre-stretching was significantly smaller than that at post-stretching (p < 0.01, 95% CI of 1.48–7.04), 10 minutes (p < 0.01, 95% CI of 2.61–10.18), and 20 minutes (p < 0.01, 95% CI of 1.88–10.59). There was no significant difference between interventions in pre-stretching (p = 0.97, 95% CI of -6.84–6.55), post-stretching (p = 0.21, 95% CI of -3.03–13.37), 10 minutes (p = 0.43, 95% CI of -12.10–5.29), or 20 minutes (p = 0.40, 95% CI of -12.64–5.17).

### Passive torque at end ROM

There was no significant two-way interaction effect (p = 0.76, partial eta squared = 0.02, F = 0.39) and no main effect for intervention (p = 0.73, partial eta squared < 0.01, F = 0.13), but there was a significant main effect for time (p < 0.01, partial eta squared = 0.33, F = 12.51) (Fig 2). For both intensities, the passive torque at pre-stretching was significantly smaller than that at post-stretching (p < 0.01, 95% CI of -6.60 –-1.62), 10 minutes (p < 0.01, 95% CI of -6.28 –-1.85), and 20 minutes (p < 0.01, 95% CI of -6.47 –-0.62) (Fig 2).

### Muscle-tendon unit stiffness

There was a significant main effect for time (p < 0.01, partial eta squared = 0.19, F = 6.08) but no main effect for intervention (p = 0.28, partial eta squared = 0.04, F = 1.21). There was a significant two-way interaction effect (intervention × time, p = 0.02, partial eta squared = 0.19, F = 3.55) (Fig 2). In the 100%POD, there was no significant difference in the muscle-tendon unit stiffness between pre-stretching and post-stretching (p = 1.00, 95% CI of -0.06–0.11), 10 minutes (p = 1.00, 95% CI of -0.07–0.13), and 20 minutes (p = 1.00, 95% CI of -0.14–0.15) (Fig 2). However, in the 120%POD, the muscle-tendon unit stiffness at pre-stretching was significantly larger than that at post-stretching (p < 0.01, 95% CI of 0.06–0.23), 10 minutes (p < 0.01, 95% CI of 0.06–0.26), and 20 minutes (p < 0.01, 95% CI of 0.02–0.30). There was no significant difference between interventions in pre-stretching (p = 0.69, 95% CI of -0.20–0.13), post-stretching (p = 0.23, 95% CI of -0.05–0.22), 10 minutes (p = 0.10, 95% CI of -0.02–0.21), or 20 minutes (p = 0.09, 95% CI of -0.27–0.02).

### NRS

There was a significant main effect for time (p < 0.01, partial eta squared = 0.71, F = 63.1) but no main effect for intervention (p < 0.01, partial eta squared = 0.40, F = 17.17). There was a significant two-way interaction effect (intervention × time, p < 0.01, partial eta squared = 0.41, F = 18.26) (Table 1). In the 100%POD, NRS at first repetition was significantly higher than that at post-stretching (p = 0.01, 95% CI of 0.27–2.59). In the 120%POD, NRS in the first (p < 0.01, 95% CI of 0.19–1.53) and second repetitions (p < 0.01, 95% CI of 3.49–5.80) were significantly higher than that at post-stretching. In the first (p < 0.01, 95% CI of 1.74–4.54) and second (p < 0.01, 95% CI of 1.21–4.07) repetitions, NRS in the 120%POD were significantly higher than those in the 100%POD.

**Table 1:** Pain of the hamstrings.

	First repetition	Second repetition	Post-measurement
100%POD	1.5 (0–2) *	1.0 (0–1.8)	0 (0–0)
120%POD	4.5 (4.0–5.0) **^,^ ^†^^,^ ^$^	4.0 (3.0–4.0) **^,^ ^$^	0 (0–0)

Data were represented as median (interquartile range).

* p < 0.05 vs. value in the 100%POD at post-measurement.

** p < 0.01 vs. value in the 120%POD at post-measurement.

^†^ p < 0.05 vs. value in the 120%POD at second repetition.

^$^ p < 0.01 vs. value in the 100%POD at the same time.

## Discussion

The present study examined the time course of changes in the flexibility of the hamstrings after static stretching at two different intensities (100%POD and 120%POD). In the 100%POD, the knee extension ROM and passive torque at end ROM increased after static stretching, and the changes continued for at least 20 minutes. On the other hand, in the 120%POD, the knee extension ROM and passive torque at end ROM increased and the muscle-tendon unit stiffness decreased after static stretching, and the changes continued for at least 20 minutes. This is the first study to investigate the time course of changes in the flexibility of the hamstrings after high-intensity static stretching, and the results indicated that the effects of stretching continued for 20 minutes.

In the present study, the knee extension ROM increased after static stretching at the intensity of both 100%POD and 120%POD. The increment in the knee extension ROM after static stretching was caused mainly by an increment in stretching tolerance [3–6] or decrease in muscle-tendon unit stiffness [7–10,21]. The present study measured the passive torque at end ROM to evaluate the change in the stretching tolerance [6,17,18]. The passive torque at end ROM increased in both intensities and the change was similar, but the muscle-tendon unit stiffness decreased only in the 120%POD, not in the 100%POD. Previous studies reported that the knee extension ROM increased immediately after static stretching at the intensity of 100%POD [36,37], but 180 seconds of stretching was required to decrease the muscle-tendon unit stiffness of the hamstrings [7,8]. On the other hand, it was reported that the knee extension ROM and passive torque at end ROM increased and the muscle-tendon unit stiffness decreased after static stretching at the intensity of 120%POD even if the stretching duration was 20 seconds or less [17,18]. Taken together, it was indicated that in the 100%POD, the knee extension ROM could increase through increasing stretching tolerance, but not the muscle-tendon unit stiffness of the hamstrings. On the other hand, in the 120%POD, the knee extension ROM could increase through both increasing stretching tolerance and decreasing the muscle-tendon unit stiffness of the hamstrings.

Several previous studies investigated the time course of changes in the flexibility of the hamstrings [28] and triceps surae [9,10,29–32] after static stretching at the intensity of 100%POD. Mizuno et al. [10] investigated the time-course effect of 5 minutes of static stretching on the triceps suare, and reported that the increased ROM after stretching returned to baseline within 60 minutes, but the decreased muscle stiffness returned to baseline within 15 minutes. For the hamstrings, Hatano et al. [28] reported that the increase in the knee extension ROM and decrease in the muscle-tendon unit stiffness after 5 minutes of static stretching continued for 30 and 20 minutes, respectively. To our best knowledge, there is no study that has examined the time course of changes in the flexibility of the hamstrings after 60 seconds of static stretching at the intensity of 100%POD. Our results showed that the increased knee extension ROM and passive torque continued for 20 or more minutes after 60 seconds of static stretching at the intensity of 100%POD, but there were no significant time course changes in muscle-tendon stiffness after stretching, consistent with previous studies showing that a stretching duration of 180 seconds is needed to decrease the stiffness [7,8].

In the results of the present study, it was not possible to directly examine the association between the stretching intensity and time course of change in the muscle-tendon unit stiffness of the hamstrings because the stiffness did not change after 60 seconds of static stretching at the intensity of 100%POD. Ryan et al. reported that static stretching with a longer duration has longer lasting effects [31]. Previous studies reported that the effects of static stretching at the intensity of 100%POD for 3–5 minutes on the muscle-tendon unit stiffness continued for 5–20 minutes [28–32]. On the other hand, the results of the present study showed that the effects of 60 seconds of static stretching at the intensity of 120%POD on the muscle-tendon unit stiffness of the hamstrings continued for at least 20 minutes. These results indicated that 60 seconds of high-intensity static stretching (120%POD) may have a lasting effect as long as, or longer than that of, 3–5 minutes of normal-intensity static stretching (100%POD), and the effects may last for at least 20 minutes.

In the present study, the median values of NRS during static stretching were 1.0–1.5 and 4.0–4.5 in the 100%POD and 120%POD, respectively, but the pain disappeared after the post-stretching measurement. These data indicated that high-intensity static stretching used in the present study was as safe as previous studies [16–18]. All studies on high-intensity static stretching [16–18], including the present study, have been conducted in healthy young adults. Therefore, it is necessary to confirm the safety of high-intensity static stretching for persons with a history of muscle-tendon injuries and the elderly.

There were some limitations. Firstly, the present study examined the time course of changes in the flexibility of the hamstrings 20 minutes after high-intensity static stretching, because previous studies reported that the decrement in the stiffness continued maximally for 20 minutes even if a longer stretching duration was used (3–5 minutes) [9,10,28–32] compared to the present study (1 minute). However, the decrement in the muscle-tendon unit stiffness after high-intensity static stretching continued for 20 or more minutes. Therefore, it is not clear when the effects of high-intensity static stretching disappear. Secondly, the present study examined the effects of 60 seconds of high-intensity static stretching. However, Ryan et al. [31] reported that a longer duration of static stretching had longer lasting effects. Therefore, it is necessary to examine the effects of duration of high-intensity static stretching on the time course of changes in the flexibility of the hamstrings to develop effective stretching techniques. Finally, the present study included participants who did not regularly perform any flexibility and strength training. Therefore, it is necessary to examine the time course of changes in flexibility after high intensity static stretching in athletes.

## Conclusions

The present study examined the time course of changes in the flexibility of the hamstrings after of two different intensities of static stretching (100%POD and 120%POD intensity) for 60 seconds. The results showed that the knee extension ROM and passive torque at end ROM increased in both intensities, and the effects continued for at least 20 minutes after stretching regardless of stretching intensity. However, the muscle-tendon unit stiffness of the hamstrings decreased only after static stretching at the intensity of 120%POD, and the effects continued for at least 20 minutes after stretching.

## Supporting information

S1 File (PDF)
